# Gut Microbiota and Acute Central Nervous System Injury: A New Target for Therapeutic Intervention

**DOI:** 10.3389/fimmu.2021.800796

**Published:** 2021-12-24

**Authors:** Bin Yuan, Xiao-jie Lu, Qi Wu

**Affiliations:** ^1^ Department of Neurosurgery, The Affiliated Wuxi No. 2 Hospital of Nanjing Medical University, Wuxi, China; ^2^ Department of Neurosurgery, The Affiliated Hospital of Jiangnan University, Wuxi, China; ^3^ Department of Neurosurgery, Jinling Hospital, Nanjing University, School of Medicine, Nanjing, China

**Keywords:** gut microbiota, stroke, traumatic brain injury, spinal cord injury, gut-brain axis

## Abstract

Acute central nervous system (CNS) injuries, including stroke, traumatic brain injury (TBI), and spinal cord injury (SCI), are the common causes of death or lifelong disabilities. Research into the role of the gut microbiota in modulating CNS function has been rapidly increasing in the past few decades, particularly in animal models. Growing preclinical and clinical evidence suggests that gut microbiota is involved in the modulation of multiple cellular and molecular mechanisms fundamental to the progression of acute CNS injury-induced pathophysiological processes. The altered composition of gut microbiota after acute CNS injury damages the equilibrium of the bidirectional gut-brain axis, aggravating secondary brain injury, cognitive impairments, and motor dysfunctions, which leads to poor prognosis by triggering pro-inflammatory responses in both peripheral circulation and CNS. This review summarizes the studies concerning gut microbiota and acute CNS injuries. Experimental models identify a bidirectional communication between the gut and CNS in post-injury gut dysbiosis, intestinal lymphatic tissue-mediated neuroinflammation, and bacterial-metabolite-associated neurotransmission. Additionally, fecal microbiota transplantation, probiotics, and prebiotics manipulating the gut microbiota can be used as effective therapeutic agents to alleviate secondary brain injury and facilitate functional outcomes. The role of gut microbiota in acute CNS injury would be an exciting frontier in clinical and experimental medicine.

## Introduction

Acute injuries to the central nervous system (CNS), such as stroke, traumatic brain injury (TBI), spinal cord injury (SCI), are critical global health problems that result in lifelong disabilities or death, leading to catastrophic changes to the injured individuals, alongside their family and even the entire community (1, 2). The processes secondary to acute CNS injuries involve a sequence of complex pathophysiological mechanisms, including excitotoxicity, electrolyte imbalance, oxidative stress, inflammation, apoptosis, pyroptosis, ferroptosis, autophagy, and cerebral edema (3). These cellular and molecular damages exacerbate neuronal cell death. Although some preclinical researchers have made many efforts to develop efficacious treatment strategies, patients with severe acute CNS injuries still have a poor prognosis. Given the prevalence of acute CNS injury-induced disabilities or death, exploring a novel and effective therapeutic regimen is imperative. In addition, a growing body of studies has shown that gut microbiota plays a pivotal role in health and disease in the host, particularly in the CNS (4–7).

Gut microbiota refers to the assemblage of bacteria, archaea, viruses, and eukaryotic microbes that colonize in the digestive tract (8). The gut microbiota contains trillions of microorganisms, over 1000 different species of known bacteria, and approximately 100 ~ 150-fold more genes than the human genome (9). At the phylum level, the gut microbiota primarily consists of Firmicutes, Bacteroidetes, Actinobacteria, Proteobacteria, and Verrucomicrobia (10). Thereinto, Firmicutes, and Bacteroidetes comprise about 90% of all the bacteria (11). Additionally, the composition of gut microbiota among individuals was influenced by diet, age, gender, environment, and genes (12). Although the microbiome is spatially restricted to the gut, it has been shown to regulate the functions of distant organs (13). Notably, advances in the sequencing of gut microbiota have revealed the close correlation between the complex ecosystem and CNS (14). Previous studies focused on exploring the bidirectional communication pathways between gut microbiota and CNS, termed the “microbiota-gut-brain” axis (MGBA) (15). The bidirectional communication pathways between the CNS and gut microbiota involve immunological, endocrine, metabolic, and neural pathways (16, 17). Recent findings have implicated that MGBA partakes in the pathogenesis of many neurological disorders such as neurodegenerative diseases(e.g., Alzheimer’s disease and Parkinson’s disease), neurodevelopmental and neuropsychiatric diseases (e.g., anxiety, depression, autism, and schizophrenia), autoimmune disease (e.g., multiple sclerosis), and acute CNS injuries (e.g., stroke, TBI and SCI) (7, 15, 18–20). In this review, we provide an update on the link between gut microbiota and acute CNS injuries.

## Microbiota-Gut-Brain Axis

### Top-Down Signaling: Brain-to-Gut

**Figure 1:**
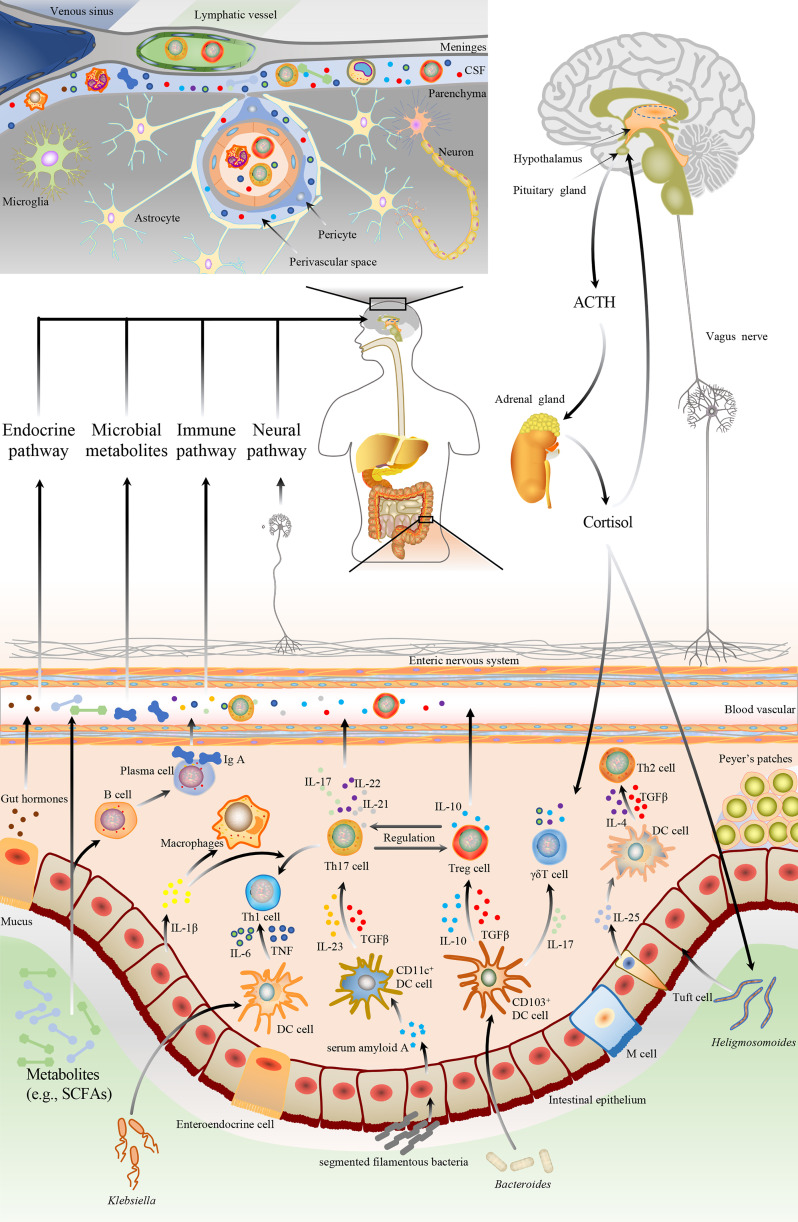
The bidirectional communication pathways between the gut microbiota and brain. The gut microbiota could bi-directionally communicate with the brain through multiple pathways, including neuronal and non-neuronal. The brain regulates the gut microbiota *via* neuronal pathways (e.g., autonomic nervous system and enteric nervous system), hypothalamic-pituitary-adrenal axis, etc. Neuronal pathways release neurotransmitters to modulate gut motility, gut barrier permeability, fluid maintenance, resident immune cell activation, and gut microbiota composition. HPA also releases cortisol to regulate gut homeostasis. Additionally, gut microbiota affects the development and pathophysiology of the brain by immunological, endocrine, metabolic, and neural pathways. Microbiomes and their metabolites could modulate the brain and behavior by affecting intestinal epithelial cells to alter gut barrier function, enteroendocrine cells to secret hormones, as well as dendritic cells and macrophage, to regulate immune and microglia activation. Gut microbiota can modulate the CD4^+^ T cells differentiation through epithelial cells or DC cells-mediated signals. ① Ectopic colonizing microbes, such as Klebsiella, can invade intestinal epithelium and stimulate DC cells to secrete proinflammatory cytokines, including IL-6 and TNF, which drive the polarization of Th1cells. ② SFB promotes Th17 polarization *via* epithelial cell-mediated CD11c^+^ DC cells activation. Epithelial cells release serum amyloid A to activate CD11c^+^ DC cells, leading to the TGF-β, IL-12, and IL-23 secretion. ③ Resident microbes, such as Bacteroides, modulate Treg cells generation by TGF-β and IL-10, which are secreted by CD103^+^ DC cells. CD103^+^ DC cells also can release IL-17 to promote γδT cell polarization. ④ Microbiota-associated Th2 cell polarization is correlated with parasite colonization such as Heligmosomoides, mediated by tuft cells secreting IL-25 to DC cells. Then activated DC cells release IL-4 and TGF-β to drive Th2 polarization. DC, dendritic cell; IL, interleukin; TNF, tumor necrosis factor; Th, T helper; TGF-β, transforming growth factor-β; SFB, segmented filamentous bacteria; SCFA, short-chain fatty acid; Treg, regulatory T cell.

In top-down signaling, the pathways involve the autonomic nervous system, enteric nervous system, the hypothalamic-pituitary-adrenal (HPA) axis, and immunological pathway (**Figure 1**). The autonomic nervous system regulates intestinal homeostasis, and the neurotransmitters, released by the activated sympathetic and parasympathetic nerve fibers, modulate gut motility, gut barrier permeability, fluid maintenance, bile secretion, resident immune cell activation, and gut microbiota makeup (21). The enteric nervous system is also responsible for gut functions, such as gut motility and fluid maintenance, a neuronal connection between the microbiota and the host. Moreover, the HPA axis is one of the vital nonneuronal transmission pathways within the MGBA, releasing cortisol to influence gut homeostasis in response to various stimuli. With the discovery of meningeal lymphatic vessels, the brain is no longer an immune-privileged site. Functional meningeal lymphatic vessels lined mainly in the dorsal part of the skull are responsible for the clearance of cerebrospinal fluid and drainage of immune cells and the periphery, communicating with the host organs (22).

### Bottom-Up Signaling: Gut-to-Brain

Two different mechanisms involved in the bottom-up signaling are neuronal and nonneuronal pathways (**Figure 1**). The vagus nerve, composed of both afferent and efferent fibers (80% vs. 20%), plays a pivotal role in bidirectionally transmitting vital information between the gut and brain. The afferent fibers stimulated by microbial metabolites and enteroendocrine neuropeptides convey hypothalamic neurons that promote pituitary secretions. In addition, the interaction between the gut and brain primarily relies on the nonneuronal pathway. Singh et al. reported that the gut microbiota-mediated neuroprotection was absent in lymphocyte-deficient mice after an experimental stroke of permanent distal middle cerebral artery occlusion (MCAO), indicating that the gut communicate with the brain by immunological pathway (23). In a transient MCAO model, intestinal CD45^+^ and CD11c^+^ cells significantly migrated from the gut to the brain and meninges at 3 days post-stroke (24). A study of a thoracic level 9 contusion SCI also showed that commercial probiotics (VSL#3) feeding triggered a protective immune response in gut-associated lymphatic tissues (GALTs) and conferred neuroprotection with the improvement of locomotor recovery after SCI (25). TBI-induced leaky gut released lipopolysaccharide (LPS), a toxic bacterial component, into the circulation that exacerbated neuroinflammation by activating microglia (26).

The MGBA is critical to the development of the human central nervous system. A prospective longitudinal study conducted by Carlson et al. investigated the correlation between gut microbial composition and cognitive ability in 89 infants. It revealed that 2-year-old cognitive function assessed with the Early Learning Composite of Mullen Scale was significantly correlated with the gut microbiota composition at one year (27). Infants with a relatively high abundance of Bacteroides showed higher performance, while those with a relatively high quantity of Faecalibacterium had a lower performance. In another clinical study of 39 infants, the α-diversity of gut microbiota was associated with functional connectivity between the amygdala and thalamus, between the anterior cingulate cortex and anterior insula, and between the supplementary motor area and the inferior parietal lobule (28). Additionally, the functional connectivity was also related to 2-year-old cognitive outcomes. These studies have demonstrated that the gut microbiota significantly affects neurodevelopment in the early stage through the MGBA.

Microbial components and metabolites such as lipopolysaccharide (LPS), long-chain fatty acids (LCFAs), short-chain fatty acids (SCFAs), trimethylamine-N-oxide (TAMO), tryptophan, and polysaccharide A (PSA) are considered to induce neuroinflammation and modulate the function of CNS either directly or by activating migration of peripheral immune cells to the brain. Although this regulation of immune cells by the microbiota occurs in the gut, peripheral immune cells could also migrate to the brain meninges and modulate the brain function (29).

## Gut Microbiota and Immunomodulation

### Gut Microbiota

Previous studies have shown that the gut microbiota-host interaction contributes to the maturation and modulation of the host immune system. Through constant contact with the gut microbiota, immune cells and epithelial cells located in the gut have achieved a homeostatic state and promote tolerogenic responses to the host commensal microbes. Under physiological conditions, all types of immune cells such as T lymphocytes, B lymphocytes, macrophages, dendritic cells (DCs), etc., counterbalance each other to preserve the host homeostasis. T helper (Th) cells and regulatory T (Treg) cells are a requisite component of the host immune system, especially in the gut-associated immune responses. Compared with germ-free (GF) mice, conventional specific-pathogen-free (SPF) mice had a more significant proportion of CD4^+^ T cells (30). Th1 cells were polarized by DCs-secreted pro-inflammatory cytokines such as interleukin(IL)-6 and IL-12 stimulated by Klebsiella (31). Parasites, such as Heligmosomoides, could activate Th2 cells through DCs-derived transforming growth factor-β(TGF-β) and IL-4 (32). Besides, segmented filamentous bacteria drive Th17 polarization *via* activation of CD11c^+^ DCs (33). The activated Th17 cells secrete high-affinity IL-17 and promote IgA transportation, memory CD4^+^ T cell differentiation, and mucin production (33). Bacteroides fragilis stimulate regulatory CD4^+^ T cells to make themselves colonize the intestinal epithelium and induce immunosuppression, mediated by CD103^+^ DCs (34). γδ T cells, another innate immune cell population in the gut epithelium, are vital for gut homeostasis regulation and pathological reaction. CD103^+^ DCs activated by gut microbiota also communicate with IL-17^+^ γδ T cells *via* cell-to-cell contact or different cytokines (35). γδ T cells protect the host from intestinal barrier damage and pathogenic bacterial invasion and exert pro-inflammatory or anti-inflammatory effects depending on the milieu (36). Additionally, the development of intestinal mucosa B cells is regulated by commensal microbes, which promote antibody production and control the expression of a differentiation-related gene through enhancement of fatty acid synthesis, glycolysis, and oxidative phosphorylation (37). Commensal microbiota-induced secretion of IL-1β by mucosal macrophages is closely correlated with the development of steady-state Th17 cells (38)(**Figure 1**).

### Microbial Components and Metabolites

Gut microbiota-derived small molecules are inextricably linked with the crosstalk between gut microbiota and host. Moreover, there is now a considerable body of experimental evidence that some metabolites of the intestinal microbiota can participate in the modulation of inflammatory cytokine production and immune cell differentiation. SCFAs produced by gut microbiota such as Faecalibacterium prausnitzii, Roseburia intestinalis, and Anaerostipes butyraticus regulate the activation and differentiation of immune cells (e.g., neutrophils, macrophages, DCs, and T cells), mediated by inhibiting histone deacetylases (HDACs) as well as activating G-protein-coupled receptors (GPCRs) (e.g., GPR41, GPR43, and GPR109A) and olfactory receptor-78 (Olfr-78) (39–41). TMAO is another gut microbiota metabolite oxidized from trimethylamine (TMA) generated by hepatic flavin-containing monooxygenases (FMOs) from dietary nutrients such as choline and L-carnitine. TAMO regulates pro-inflammatory responses *via* activating the NOD-like receptor family, pyrin domain-containing protein 3 (NLRP3) inflammasome, mitogen-activated protein kinase (MAPK), and nuclear factor-κB (NF-κB) signaling pathway (42). Additionally, tryptophan has also been demonstrated to be involved in the functional modulation of intestinal intraepithelial lymphocytes and innate lymphocytes *via* activating the aryl hydrocarbon receptor (AHR) (43). PSA, produced by Bacteroides fragilis, activates toll-like receptor 2(TLR2) expressed on DCs and Treg cells, triggering anti-inflammatory cytokine IL-10 (43). PSA also regulates the differentiation of naive CD4^+^ T cells into Th1 cells, skewing Th1/Th2 ratio towards Th1 cells (44). Interestingly, a study found that CD39 expression is required for Treg cells to migrate to the CNS, depending on PSA-driven effects on the Treg cells (45).

## Gut Microbiota and Acute CNS Injuries

### Stroke

Stroke is the second leading cause of death worldwide. Morbidity and mortality of stroke grow in many countries, contributing to financial burden and loss of life quality, and thus diminishing national happiness index. Approximately 15 million people around the world are victims of a stroke every year (46). There are two types of strokes: ischemic stroke and hemorrhagic stroke. Recent evidence shows that shifts in the gut microbial composition occur rapidly after stroke in clinical and experimental studies. Therefore, alteration of the gut microbiota directly regulates the reactions secondary to brain injury through central and peripheral immunity and indirectly determines the brain’s sequel to these types of catastrophic events.

#### Ischemic Stroke

Reportedly, ischemic stroke accounts for ~80% of all strokes (47), and the gut microbiota plays an essential role in the pathogenesis and prognosis of ischemic stroke. Multiple studies have shown that ischemic stroke significantly changes the gut microbiota composition (21, 23, 48–66). These studies have been summarized in **Table 1**. Although dysbiosis of the gut microbiota has been proved in previous studies, controversy still exists on the specific microbiota difference. Compelling evidence has been identified that confounding factors such as age, diet, behavior, antibiotic use, prolonged stress, environment, and genetics compose the gut microbiota, which may be influenced by the contradictory results of the above studies. Thus, more studies are needed to clarify the role of gut microbiota dysbiosis in the pathogenesis and prognosis of ischemic stroke. Recently, a preclinical study also suggested that the alteration in the gut microbiota was associated with hemorrhagic transformation (HT) (71). The relative abundance of Proteobacteria and Actinobacteria was significantly increased in HT rats after experimental stroke, indicating that the gut microbiota is involved in the progression of ischemic stroke.

**Table 1:** A summary of preclinical and human studies on the gut microbiota and ischemic stroke.

	Subjects	Methods	Key findings
Yin J, et al. (2015) (48)	322 patients vs. 231 controls	16S rRNA (V4) sequencing & LC-MS	Patients with stroke and transient ischemic attack presented the gut microbiota dysbiosis, which increased Enterobacter, Megasphaera, Oscillibacter, and Desulfovibrio and decreased Bacteroides, Prevotella, and Faecalibacterium. Patients with stroke and the transient ischemic attack had lower trimethylamine N-oxide (TMAO) compared with asymptomatic patients.
Stanley D, et al. (2016) (52)	36 patients vs. 9 hospital-based controls vs. 10 healthy controls; middle cerebral artery occlusion (MCAO) mice	16S rRNA sequencing	Common commensal bacteria resided in the intestinal tracts contributed to the post-stroke infections in patients with ischemic stroke. This was also observed in a mouse model of ischemic stroke. In the experimental stroke, post-stroke infection was only seen in specific pathogen-free (SPF) mice, not germ-free (GF) mice.
Nie J, et al. (2018) (51)	622 patients vs. 622 controls	LC-MS	The increment of serum TMAO level increased the risk of the first stroke. Patients with higher TMAO levels (≥1.79 μmol/L) had a significantly higher risk of the first stroke.
Zeng X, et al. (2019) (50)	141 patients	16S rRNA sequencing & GS-MS	Compared with the low-risk group, opportunistic pathogens (e.g., Enterobacteriaceae and Veillonellaceae) and lactate-producing bacteria (e.g., Bifidobacterium and Lactobacillus) were increased, as well as butyrate-producing bacteria (e.g., Lachnospiraceae and Ruminococcaceae) were decreased in the high-risk group. The fecal butyrate concentrations in the high-risk group were lower than those in the low-risk group. Moreover, the concentrations of other short-chain fatty acids (SCFAs) (e.g., acetate, propionate, isobutyrate, isovalerate, and valerate) in the feces were significantly different between the three groups.
Haak BW, et al. (2020) (49)	349 patients vs. 51 controls	16S rRNA (V3-V4) sequencing & LC-MS	The TMAO level in stroke patients was two-fold lower than that of the healthy controls. Lower abundance of butyrate-producing bacteria within 24h of hospital admission was an independent predictor of enhanced risk of post-stroke infection, but not of mortality or functional patient outcome.
Xu DJ, et al. (2021) (60)	61 large artery atherosclerotic (LAA) stroke vs. 20 cardioembolic (CE) stroke vs. 51 asymptomatic controls	16S rRNA (V4-V5) sequencing & LC-MS	The TMAO levels in the plasma of patients with LAA and CE strokes were significantly higher than those in controls. Moreover, the plasma TMAO level in the LAA stroke patients was positively associated with the carotid plaque area. The composition and the function of gut microbiota in the patients with LAA stroke were significantly different from those in the asymptomatic controls. In contrast, no significant difference between CE stroke patients and the asymptomatic controls was observed in the present study.
Ling Y, et al. (2020) (61)	53 patients with post-stroke cognitive impairment (PSCI) vs. 40 patients with post-stroke non-cognitive impairment (PSNCI)	16S rRNA (V3-V4) sequencing	Compared with the patient with PSNCI, the abundance of Proteobacteria was highly increased in the patients with PSCI. The abundances of Clostridia, Clostridiales, Lachnospiraceae, and Lachnospiraceae_other were significantly decreased in the patients with PSCI after adjusting to age. The Kyoto Encyclopedia of Genes and Genomes analysis showed the progressive enriched module for folding, sorting, and degradation (chaperones and folding catalysts) and the significantly decreased modules related to metabolisms of cofactors and vitamins, amino acid, and lipid in patients with PSCI.
Xiang L, et al. (2020) (62)	20 patients vs. 16 controls	16S rRNA (V3) sequencing	Stroke patients had fewer Firmicutes than controls. Two optimal bacterial species, Lachnospiraceae (OTU_45) and Bacteroides served as markers of lacunar infarction. Two optimal bacterial species, Bilophila and Lachnospiraceae (OTU_338)), served as markers of non-lacunar acute ischemic infarction. Three optimal bacterial species, Pseudomonas, Sphingomonadaceae, and Akkermansia, served as markers of post-ischemic stroke patients with 15 days of treatment.
Tan C, et al. (2021) (67)	140 patients vs. 92 controls	16S rRNA (V4) sequencing & GS-MS	Patients with acute ischemic stroke are characterized by a lack of SCFAs-producing bacteria (Roseburia, Bacteroides, Lachnospiraceae, Faecalibacterium, Blautia, and Anaerostipes) and an overload of Lactobacillaceae, Akkermansia, Enterobacteriaceae, and Porphyromonadaceae in their feces. The SCFAs levels were negatively related to stroke severity and prognosis. Reduced fecal SCFAs level, especially acetate, was correlated with an increased risk of 3-month unfavorable outcomes.
Zhang J, et al. (2021) (68)	351 patients vs. 150 controls	LC-MS	Patients with an unfavorable outcome had significantly increased plasma TMAO levels on admission. Plasma TMAO levels on admission were an independent predictor of functional outcome and mortality after acute ischemic stroke.
Guo Q, et al. (2021) (69)	49 patients vs. 30 controls	16S rRNA (V3-V4) sequencing	The acute ischemic stroke patients treated with Tanhuo Decoction had a better outcome than the controls on both clinical outcome and gut microbiota characteristics. Tanhuo Decoction treatment significantly decreased the lipopolysaccharide (LPS)-producing bacteria (Bacteroidaceae and Bacteroides) to reduce LPS biosynthesis. The acute ischemic stroke patients treated with Tanhuo Decoction also exhibited the potential to decrease the biosynthesis of trimethylamine (TMA), the precursor of TMAO, and increase TMA’s degradation.
Huang Y, et al. (2021) (63)	76 patients vs. 19 controls	16S rRNA (V3-V4) sequencing	Stroke patients had a significantly higher abundance of Enterococcus and lower abundances of Bacteroides, Escherichia-Shigella, and Megamonas. Compared with stroke patients, patients with post-stroke cognitive impairment had a significantly higher proportion of Enterococcus, Bacteroides, and Escherichia-Shigella and a lower content of Faecalibacterium. Patients with the post-stroke affective disorder had a significantly higher proportion of Bacteroides and Escherichia-Shigella and a lower proportion of Enterococcus and Faecalibacterium.
Sun T, et al. (2021) (70)	953 patients vs. 953 controls	LC-MS/MS	Plasma TMAO levels in patients with ischemic stroke were significantly increased. Higher plasma TMAO levels were correlated with increased odds of ischemic stroke. The adjusted odds ratios for ischemic stroke per 1 μmol/L increase of plasma TMAO was 1.05.
Xu K, et al. (2021) (64)	Cohort 1: 28 patients vs. 28 controls; Cohort 2: 124 patients; MCAO mice	16S rRNA sequencing	Enriched Enterobacteriaceae was an independent risk factor for acute ischaemic stroke patients in early-stage recovery. MCAO mice showed rapid gut dysbiosis with Enterobacteriaceae blooming, associated with intestinal ischemia and nitrate production. Enterobacteriaceae exacerbates brain infarction by accelerating LPS/toll-like receptor 4(TLR4)-mediated systemic inflammation. Inhibiting Enterobacteriaceae overgrowth by diminishing nitrate generation or inhibiting nitrate respiration alleviates brain infarction.
Houlden A, et al. (2016) (21)	MCAO mice	16S rRNA PCR	The alteration of the caecal microbiota composition following stroke could be mediated by noradrenaline release from the autonomic nervous system, changing caecal mucoprotein production and goblet cell numbers. Specific changes in Peptococcaceae and Prevotellaceae after stroke were correlated with the severity of the injury.
Singh V, et al. (2016) (54)	MCAO mice	16S rRNA (V1-V3) sequencing	Reduced species diversity and bacterial overgrowth of bacteroidetes were associated with intestinal barrier dysfunction and reduced intestinal motility. GF mice recolonized with poststroke gut microbiota exacerbates infarct volume and functional deficits following stroke, mediated by the migration of intestinal pro-inflammatory T cells to the ischemic brain. Fecal microbiota transplantation (FMT) could normalize brain lesion-induced dysbiosis and improve stroke outcomes.
Benakis C, et al. (2016) (55)	MCAO mice	16S rRNA (V4-V5) sequencing	Antibiotic-induced alterations in the gut microbiota reduced brain injury after ischemic stroke. Dysbiosis following ischemic stroke changed intestinal immune homeostasis, leading to an increase in regulatory T(Treg) cells and a reduction in IL-17+ γδ T cells through altered dendritic cell activity. Moreover, dysbiosis blocked the migration of effector T cells from the gut to the leptomeninges.
Winek K, et al. (2016) (59)	MCAO mice	–	Microbiota-depleted mice stopped the antibiotic cocktail pretreatment 3 days before surgery significantly decreased survival after MCAO. Microbiota-depleted animals treated by continuous antibiotic treatment or colonized with SPF microbiota before surgery rescued from severe acute colitis.
Spychala MS, et al. (2018) (53)	MCAO mice	16S rRNA (V4-V5) sequencing	The Firmicutes to Bacteroidetes ratio in aged mice increased ∼9-fold compared to young. The gut microbiota in the young manipulated by fecal from aged mice increased mortality, decreased behavioral performance, and increased cytokine levels following MCAO, altering the microbiota in the aged by fecal gavage to resemble that of young increased survival and improved recovery following MCAO.
Singh V, et al. (2018) (23)	MCAO mice	16S rRNA (V1-V3) sequencing	Bacterial colonization reduces stroke volumes by increasing cerebral expression of cytokines and microglia/macrophage cell counts. The gut microbiota-mediated neuroprotection was absent in lymphocyte-deficient mice.
Benakis C, et al. (2020) (56)	MCAO mice	16S rRNA (V4) sequencing	Single antibiotic treatment with either ampicillin or vancomycin, but not neomycin, significantly reduced the infarct volume and improved motor sensory function 3 days after stroke. Bacteroidetes S24.7 and the enzymatic pathway for aromatic metabolism were correlated with infarct size. The gut microbiota composition in the ampicillin-treated mice was associated with reduced gut inflammation, a long-term favorable outcome, and a reduction of brain tissue loss. Regulation of SCFAs and tryptophan pathways induced by ampicillin could be predictive of stroke outcomes.
Sadler R, et al. (2020) (57)	MCAO and photothrombotic (PT) mice	GC-MS	SCFAs supplementation in the drinking water significantly improved recovery of limb motor function by altering contralesional cortex connectivity, which is related to SCFAs-dependent changes in spine and synapse densities. A substantial impact of SCFAs on microglial activation contributes to the structural and functional remodeling, mediated by the recruitment of T cells to the infarcted brain.
Lee J, et al. (2020) (58)	MCAO mice	16S rRNA (V4) sequencing & LC-MS	Aged stroke mice transplanted the young fecal improved post-stroke neurological deficits and inflammation, which correlated with higher SCFAs levels and SCFAs-producers such as Bifidobacterium longum, Clostridium symbiosum, Faecalibacterium prausnitzii, and Lactobacillus fermentum.
Jeon J, et al. (2020) (65)	MCAO pig	16S rRNA (V3-V4) sequencing	Compared with pre-stroke populations, the abundance of the Proteobacteria was significantly increased, while the abundances of Firmicutes and lactic acid bacteria Lactobacillus decreased at 3 days poststroke. The gut microbial pattern returned to similar values as prestrike at 5 days poststroke.
Benakis C, et al. (2020) (56)	MCAO mice	16S rRNA (V4) sequencing	Mice treated with a cocktail of antibiotics significantly reduced infarct volume in the acute phase of stroke. Single antibiotic treatment with either ampicillin or vancomycin, but not neomycin, significantly reduced infarct volume and improved neurological function 3 days after stroke. Bacteroidetes S24.7 and the enzymatic pathway for aromatic metabolism were associated with infarct size after stroke. The gut microbiota signature in the ampicillin-treated mice was correlated with reduced intestinal inflammation, long-term favorable outcome and was predictive of SCFAs and tryptophan pathways.
Huang Q, et al. (2021) (71)	MCAO rat	16S rRNA (V3-V4) sequencing & GC-MS	Compared with non-hemorrhagic transformation (HT) rats, the relative abundances of Proteobacteria and Actinobacteria were enriched in HT rats. Total SCFAs levels, especially butyrate and valeric acid, were significantly decreased in the cecal contents of HT rats. The rats colonized with gut microbiota from HT rats showed increased susceptibility to HT.
Zhang P, et al. (2021) (72)	MCAO mice	16S rRNA sequencing & HPLC-MS	Atorvastatin significantly ameliorated neurological defects and reduced microglia-mediated neuroinflammation after experimental stroke. Atorvastatin increased the abundance of Firmicutes and Lactobacillus, decreased Bacteroidetes abundance, increased fecal butyrate level, promoted intestinal barrier function by elevating the expression of claudin-1, occludin and mucoprotein 2, as well as regulated intestinal immune function. Transplantation of atorvastatin-treated mice fecal microbiota alleviated neuroinflammation in MCAO mice.
Huang JT, et al. (2021) (73)	MCAO mice	16S rRNA sequencing	The transplantation of gut microbiota collected from calorie-restriction-treated mice was eligible to have better long-term rehabilitation. Bifidobacterium was enriched in calorie-restriction mice. Bifidobacterium administration improved the long-term rehabilitation of stroke mice
Zhu W, et al. (2021) (74)	MCAO mice	16S rRNA (V4) sequencing & LC-MS	The human fecal microbial transplantation study showed TMAO production and stroke severity are transmissible traits. TMAO and choline supplementation exacerbated infarct size and functional impairment. Gut microbial CutC increased host TMAO levels and aggravated cerebral infarct size and functional deficits after stroke.
Wu W, et al. (2021) (66)	MACO rat	16S rRNA (V3-V4) sequencing & LC-MS	The abundance of the Firmicutes phylum was decreased, whereas Proteobacteria and Deferribacteres were increased after stroke. Ruminococcus_sp_15975 might serve as a biomarker for the stroke. Many metabolites, such as L-leucine, L-valine, and L-phenylalanine, differed between the stroke and sham groups, mainly involved in mineral absorption and cholinergic synapse pathways.
Yuan Q, et al. (2021) (75)	MCAO mice	16S rRNA sequencing & GC-MS	Lactulose supplementation significantly improved the functional outcome after stroke by downregulating inflammatory reaction and increased anti-inflammatory factors in the brain and gut. Lactulose supplementation improved intestinal barrier injury and restored gut microbiota dysbiosis after stroke.

16S rRNA, 16S ribosomal RNA.

PCR, polymerase chain reaction.

LC-MS, liquid chromatography-mass spectrometry.

GC-MS, gas chromatography-mass spectrometry.

HPLC-MS, high-performance liquid chromatography-mass spectrometry.

Mechanistically, the gut microbiota-mediated neuro-protection greatly depended on the microglia and lymphocyte responses, significantly increasing Th cells, polarized Treg cells, and Th17 cell counts in the intestinal Peyer’s patches (23). Pro-inflammatory Th1, Th17, and γδT cells often increase inflammatory damage, while Treg cells suppress post-stroke inflammation by secreting the anti‐inflammatory cytokine IL‐10. Alteration of gut microbiota following a stroke in the bacterial population triggers pro-inflammatory T cells responses, migrates intestinal immune cells to the meninges involved in secondary brain injury, and worsens stroke outcome. In GF MCAO animal models, mice transplanted with post-stroke fecal content presented increased infarct volume and functional deficits by inducing pro-inflammatory T cell polarization. Moreover, restoration of gut microbiota homeostasis with fecal microbiota transplantation (FMT) reduced infarct volume, improved stroke outcome and promoted the migration of intestinal Treg cells to the ischemic area in the brain (54). Also, intestinal dysbiosis following ischemic stroke was found to regulate immune homeostasis in the small intestine with increased Treg cells and decreased IL-17^+^ γδT cells, mediated by DCs. The neuroprotective effect of IL-10 was identified as a regulator of Treg cell-mediated IL-17^+^ γδT cell suppression (55).

Microbial-derived metabolites also correlate with the progression and prognosis of ischemic stroke. Ischemic stroke patients had significant gut microbiota dysbiosis with an increased abundance of SCFAs-producing bacteria such as Odoribacter, Akkermansia, which closely correlated with the stroke outcome (76). However, Zeng et al. reported that people with a high risk of stroke had lower levels of butyrate-producing bacteria (e.g., Lachnospiraceae and Ruminococcaceae) and fecal butyrate (50). Tan et al. also reported a lack of SCFAs-producing bacteria and a low fecal SCFAs level in acute ischemic stroke patients (67). Moreover, the reduced fecal SCFAs were correlated with an increased risk of 3-month unfavorable outcomes (67). The differences in the results of these clinical studies may be due to the small cohorts of the studies. These findings need to be further validated by higher-quality clinical studies with large cohorts. In experimental stroke, reduced plasma SCFAs level correlated with a worse stroke outcome in mice, and SCFAs supplementation improved behavioral recovery with modified poststroke cortical connectivity and synaptic plasticity by recruiting T lymphocytes on modulation of microglial activation, as reflected by the increase in Treg cells (57). Oral gavage of SCFAs-producing bacteria such as Bifidobacterium longum, Clostridium symbiosum, Faecalibacterium prausnitzii, and Lactobacillus fermentum alleviated post-stroke neurological deficits and inflammation by increasing populations of Treg cells and reducing the percentage of IL-17^+^ γδT cells (58). Pretreatment with Clostridium butyricum improved neurological deficits and decreased hippocampal apoptosis by increasing butyrate and reducing brain oxidative stress in experimental stroke (77). Furthermore, Zhou et al. reported that butyrate alleviated neuronal apoptosis following stroke *via* GPR41/Gβγ/PI3K/Akt pathway (78). SCFAs could also improve outcomes by protecting gut epithelial cells against stroke-induced gut leakiness by enhancing tight junction proteins (79). Furthermore, sodium butyrate reduced infarct volume and improved neurological function Recently, Huang et al. found that the significant decrease of SCFAs in cecal contents, especially butyrate and valeric acid, was closely related hemorrhagic transformation after ischemic stroke (71).

It has been demonstrated that there is a significant association between TMAO level and various diseases, including stroke (80). Although several clinical studies have identified a correlation between TMAO level and stroke, the results remain controversial. Most studies show that the plasma TMAO concentrations in stroke patients are significantly higher than those in control patients, and its high level is positively related to the severity of the stroke (51, 68). A large-scale case-control study with 953 sex- and age-matched pairs performed by Sun et al. suggested that the plasma TMAO concentrations in patients with first acute ischemic stroke were significantly elevated (70). Furthermore, further analysis revealed that the multivariable-adjusted odds ratios for ischemic stroke per 1 μmol/L increase of plasma TMAO level were 1.05. Tan et al. reported that TMAO concentrations decreased with time after stroke, and elevated TMAO levels at an early stage predicted poor stroke outcomes (81). A meta-analysis also showed that compared with non-stroke controls, TMAO increased the stroke risks by 68% and accreted 2.201 umol/L on the average level of TMAO in stroke patients (82). Zhu et al.’s study also suggested gut microbiota can impact stroke severity *via* the gut microbial CutC-mediated TMAO pathway, which exacerbated cerebral infarct size and functional deficits (74).

The activation of the kynurenine pathway for tryptophan degradation correlates with stroke-induced inflammatory responses and unfavorable outcomes^53^. Tryptophan catabolites regulate intestinal immune cell function by activating AHR. Pharmacological and genetic inhibition of neural cell-specific AHR activation improved stroke outcomes in the MCAO mice model (83). Furthermore, tryptophan catabolism positively correlated with the severity of stroke outcome and might be associated with stroke-induced inflammatory response (51). Besides, xenobiotic/aromatic compound metabolism was a predictive marker of the size of the ischemic lesion (56).

Multiple studies have demonstrated that antibiotic-induced dysbiosis promotes the proliferation and differentiation of T cells in the gut to either improve or worsen outcomes in experimental stroke. Mice pretreated with ampicillin or vancomycin significantly improved the outcome of stroke, whose neuroprotection is related to a shift with increased Proteobacteria and Firmicutes and reduction of Bacteroidetes caused by antibiotics (56). Particularly, Bacteroidetes S24.7 was closely associated with infarct size. However, Winek et al. reported stroke mice pretreated with quintuple broad-spectrum antibiotics presented with the damaged gut epithelium and worsened outcome (59). This controversial result still needs further study to clarify. FMT is a novel and potent treatment strategy in patients with gut microbiota dysbiosis obtained from fecal microbiota in healthy individuals. FMT exerts a neuroprotective effect by altering gut microbial metabolites production and reducing pro-inflammatory gut bacteria, alleviating inflammatory response and oxidative stress in the brain. Restoring gut microbiota homeostasis with FMT from healthy donors reduced lesion size by increasing Treg cells (54). MCAO mice receiving FMT from anti-inflammatory donors reduced the infarct volume by 54% (55). Additionally, FMT from young microbiota was also beneficial to stroke recovery (53). Oral gavage of SCFAs-producing bacteria or SCFAs supplementation also alleviated neurological deficits and improved poststroke recovery by reducing IL-17^+^ γδT cells in the ischemic brain (57, 58). Recently, Zhang et al. found that atorvastatin significantly alleviated the defects in sensorimotor behaviors and reduced microglia-mediated neuroinflammation by increasing the abundance of Firmicutes and Lactobacillus, decreasing the abundance of Bacteroidetes abundance, increasing fecal butyrate level, promoting intestinal barrier function, as well as regulating intestinal immune function (reduced monocyte chemotactic protein 1(MCP-1), tumor necrosis factor-α(TNF-α) and increased IL-10) in the mice with permanent MCAO (72). Calorie restriction also can promote ischemic stroke rehabilitation *via* enriching the abundance of Bifidobacterium (73). Tanhuo decoction also promoted poststroke recovery by decreasing the biosynthesis of TMA, the precursor of TMAO, and increasing the expression of trimethylamine-corrinoid protein Co-methyltransferase (mttB), which catabolizes TMA to methane (69). Additionally, Lactulose supplementation was shown to significantly improve the functional outcome of stroke, which is possibly mediated by repairing intestinal barrier injury and improving gut microbiota dysbiosis following stroke (75).

#### Hemorrhagic Stroke

Hemorrhagic stroke includes intracranial and subarachnoid hemorrhage. Intracranial hemorrhage accounts for 80% of hemorrhagic stroke and 10-15% of all strokes, which is primarily caused by hypertension-induced small vessel rupture, while subarachnoid bleeding is mainly due to intracranial aneurysms rupture (84). Hemorrhagic stroke is characterized by high mortality and morbidity, which burdens society and families. However, there are, to date, few studies focused on the exploration of the correlation between hemorrhagic stroke and gut microbiota. A few studies reported that gut microbiota dysbiosis contributes to hypertension and intracranial aneurysms. However, the direct relationship between gut microbiota and hemorrhagic stroke has not been studied. Both clinical and animal studies are warranted in the future.

#### Gut Microbiota and Intracranial Aneurysms

A case-control metagenome-wide association study showed that the structural heterogeneity of intestinal microbiota in patients with intracranial aneurysm (IA) was significantly decreased compared to healthy controls, which had an increased abundance of Bacteroides, Parabacteroides, Ruminococcus, and Blautia in IA patients and an enrichment of Faecalibacterium, Eubacterium, Collinsella, and Lactobacillus (85). Recently, another multicenter, prospective case-control study reported that the abundance of the genus Campylobacter and Campylobacter ureolyticus was significantly higher in patients with ruptured IA than that in patients with unruptured IA, which may be associated with the rupture of IA (86). Further analysis suggested that gut microbiota promoted the pathogenesis of IA by regulating plasma amino acids (e.g., taurine, hypotaurine, L-histidine, and L-citrulline) and fatty acid levels (85). Compared to mice transplanted with healthy control feces, the incidence of IA in mice transplanted with the feces of IA patients was significantly increased (85). Mechanistically, supplementation with taurine or H.hathewayi reduces the formation and rupture of IA by blunting cerebrovascular inflammatory processes, reducing extracellular matrix remodeling, and maintaining the structural integrity of cerebral blood vessels. Similarly, Fumiaki Shikata et al. also reported that the gut microbiota contributes to the development of IA by modulating inflammation in the experimental IA model (87). Gut microbiota depletion by an oral antibiotic cocktail of ampicillin, metronidazole, neomycin, and vancomycin (AMNV) significantly reduced the incidence of IA *via* decreasing macrophage infiltration and the expression of pro-inflammatory cytokines such as IL-1β and IL-6 in vascular wells (87). These results suggest that gut microbiota is closely correlated with the development of IA. Additionally, human studies are needed to determine the exact contribution of the gut microbiota to the pathophysiology of IA and aneurysmal subarachnoid hemorrhage in humans.

### Traumatic Brain Injury (TBI)

Traumatic brain injury (TBI) is one of the most common neurological diseases, with an estimated incidence of approximately 50 million people worldwide annually, leading to thousands of deaths and disabilities (88). TBI induces various secondary progressive brain damage contributing to varied functional outcomes. TBI also influences the gut barrier integrity, gut function, and gut microbiota composition (89). In turn, gut microbiota alterations may regulate a pro-inflammatory response following TBI and aggravate secondary brain injury. However, the information on TBI-induced gut microbiota dysbiosis is scarce for now. The relevant studies are summarized in **Table 2**.

**Table 2:** A summary of preclinical and human studies on the gut microbiota and traumatic brain injury.

	Subjects	Methods	Key findings
Mahajan C, et al. (2021) (90)	101 patients	–	All organisms belonged to the Proteobacteria phylum, especially Enterobacteriaceae forming the largest group after traumatic brain injury (TBI). TBI is associated with widespread colonization with Proteobacteria as early as 48 hours after injury.
Hou Y, et al. (2021) (91)	24 patients vs. 10 controls; surgical brain injury (SBI) rat	16S rRNA sequencing & HPLC-MS	The abundances of Enterococcus, Parabacteroides, Akkermansia, and Lachnoclostridium were significantly increased, whereas the relative abundances of Bifidobacterium and Faecalibacterium were decreased in the patients with TBI. Oral administration of brain protein combined with probiotics alleviated inflammatory gut damage by affecting tryptophan-related pathways.
Treangen TJ, et al. (2018) (92)	controlled cortical impact (CCI) mice	16S rRNA (V3-V4) sequencing	At a high-level view, the abundances of Marvinbryantia and Clostridiales were significantly changed after TBI. Lactobacillus gasseri, Ruminococcus flavefaciens, and Eubacterium ventriosum were decreased at the species level, while Eubacterium sulci and Marvinbryantia formatexigens and were increased after TBI.
Li H, et al. (2018) (93)	weight-drop impact (WDI) mice	–	Clostridium butyricum treatment improved neurological deficits, brain edema, neurodegeneration, and blood-brain barrier impairment. Clostridium butyricum treatment increased tight junction proteins, p-Akt, and Bcl-2 and decreased expression of Bax. Mice treated by Clostridium butyricum showed an increased intestinal Glucagon-like peptide 1(GLP-1) secretion and upregulated the expression of cerebral GLP-1 receptor.
Simon DW, et al. (2020) (89)	CCI mice	16S rRNA (V4) sequencing	Mice receiving pretreatment of ampicillin, metronidazole, neomycin, and vancomycin(AMNV) before surgery increased CA1’s density of hippocampal neuronal and reduced Iba-1 positive cells at 72 h after TBI. Mice pretreated by AMNV alleviated associative learning deficit and decreased lesion volume after TBI.
Angoa-Pérez M, et al. (2020) (94)	WDI mice	16S rRNA (V4) sequencing	An early increase in microglial activation persisted from 0-day to 90-day post-injury, compounded by substantial increases in astrocyte reactivity and phosphorylated tau. Few differences in the microbial community were observed in mice exposed to repetitive, mild TBI (rmTBI). The progressive emergence of white matter damage and cognitive deficits following rmTBI was not associated with the altered gut microbiota.
Opeyemi OM, et al. (2021) (95)	CCI mice model	16S rRNA (V4) sequencing & HPLC-MS	Bacteria from Lachnospiraceae, Ruminococcaceae, and Bacteroidaceae families were depleted, while bacteria from the Verrucomicrobiaceae family were enriched. Fecal SCFAs such as acetate were reduced at 7 days and 28 days following TBI; SCFAs administration improved spatial learning after TBI.
Du D, et al. (2021) (96)	CCI rat	16S rRNA (V3-V4) sequencing & HPLC-MS	TBI induced significant changes in the gut microbiome, including the alpha- and beta-bacterial diversity and the microbiome composition at 8 days after TBI. Fecal microbiota transplantation (FMT) could rescue these changes and relieve neurological deficits after TBI. Metabolomics results showed that the level of trimethylamine (TMA) in feces and the level of trimethylamine N-oxide (TMAO) in the ipsilateral brain and serum was increased after TBI. At the same time, FMT decreased TMA levels in the feces and TMAO levels in the ipsilateral brain and serum. FMT can restore gut microbiota dysbiosis and relieve neurological deficits, possibly through the TMA-TMAO-methionine sulfoxide reductase A (MsrA) signaling pathway after TBI.
You W, et al. (2021) (97)	lateral fluid percussion injury mice model	16S rRNA (V3-V4) sequencing & HPLC-MS	The diversity of gut microbiota experienced a time-dependent change from 1 h to 7 days post-TBI. The decreased levels of bile acids, especially secondary bile acids, were related to intestinal inflammation after TBI. Staphylococcus and Lachnospiraceae may contribute to the bile acid metabolic changes.
Celorrio M, et al. (2021) (98)	CCI mice	PCR	Antibiotic-induced gut microbial dysbiosis significantly worsened neuronal loss after TBI. Antibiotic exposure for 1 week after TBI decreased T lymphocyte infiltration, increased microglial pro-inflammatory markers, and reduced cortical infiltration of Ly6C^high^ monocytes. Gut microbiota dysbiosis was associated with increased hippocampal neuronal loss and fear memory response 3 months after TBI.

16S rRNA, 16S ribosomal RNA.

PCR, polymerase chain reaction.

HPLC-MS, high-performance liquid chromatography-mass spectrometry.

Recently, an observational study investigated the characteristics of gut microbiota in 101 TBI patients and found that organisms from rectal swabs obtained on days 0, 3, and 7 after admission belonged to the Proteobacteria phylum, with Enterobacteriaceae forming the largest group (90). Hou et al. also analyzed the gut microbiota composition in a small cohort (10 healthy control volunteers vs. 24 TBI patients) and reported that the abundance of Enterococcus, Parabacteroides, Akkermansia, and Lachnoclostridium were significantly increased, while the abundance of Bifidobacterium and Faecalibacterium were decreased in TBI patients (91).

In the controlled cortical impact (CCI) mouse model, the gut microbiota significantly decreased in Lactobacillus gasseri, Ruminococcus flavefaciens, and Eubacterium ventriosum and increased dramatically in Eubacterium sulci and Marvinbryantia formatexigens at 24h post-CCI (92). In an experimental weight-drop injury model, the severity of TBI is correlated with the alteration in Bacteroidetes, Porphyromonadaceae, Firmicutes, and α-Proteobacteria (21). Nicholson et al. found a reduced Firmicutes/Bacteroidetes ratio in gut microbiota composition occurring at 2h post-injury was significantly related to MRI-determined lesion volume and behavioral function defects (99). In the lateral fluid percussion injury mice model, You et al. also observed the alterations of gut microbiota and bile acid profile (97). Further analysis found that specific bacterial taxa such as Staphylococcus and Lachnospiraceae could be associated with the bile acid metabolic changes, resulting in intestinal inflammation. Interestingly, Angoa-Pérez et al. found that repetitive, mild TBI did not cause alterations in the gut microbiota composition (94). Although differences in gut microbiota composition have been observed after TBI in animal models, the exact regulatory mechanism remains elusive. A study considered that vagal afferent alterations, TBI-induced increase of cholecystokinin level, might be responsible for gut dysfunction through activation of the vago-vagal NTS-inhibitory pathway (100). Additionally, in another experimental TBI, the gut upregulated the expression of glycoproteins to recruit immune cells and activate inflammatory signals, resulting in altered mucosal integrity (101). The leaky gut allowed toxic bacterial components such as LPS to enter the circulation that mediates neuroinflammation by activating microglia following TBI (26). Furthermore, the permeability of the blood-brain barrier (BBB) can increase up to 4 times more than normal within 6h following TBI. The increased BBB permeability aggravates the gut dysbiosis-induced neuroinflammation by LPS exposure, γδT cell activation, and activated microglia differentiation into the M1 phenotype. A recent preclinical study performed by Celorrio et al. suggested that antibiotic-induced gut microbial dysbiosis established before TBI significantly worsened neuronal loss, reduced cortical infiltration of Ly6C^high^ monocytes and T lymphocyte, increased microglial pro-inflammatory markers, and impaired neurogenesis after TBI (98).

CCI mice pretreated with AMNV 2 weeks before CCI presented increased neuronal density in the hippocampus at 72h post-injury, while mice treated with AMNV right after CCI showed reduced lesion volume and attenuated associative learning deficit at 22 days (89). TBI mice treated by Clostridium butyricum also improved neurological deficits, attenuates brain edema, ameliorated neurodegeneration, and alleviated BBB impairment *via* elevating intestinal Glucagon-like peptide 1(GLP-1) secretion (93). SCFAs supplementation also improved spatial learning following CCI-induced TBI, mediated by activating the neurotrophic tyrosine kinase receptor type 1 (TrkA) pathway (95, 102). Probiotic supplementation also significantly remedied the gut microbiota dysbiosis and decreased the intestinal permeability following experimental TBI by reducing the intestinal mucosa damage, alleviating brain injury (103). In human studies, probiotic treatment could relieve systemic inflammatory response, decrease nosocomial infection rate, and improve the recovery of patients with TBI (104, 105). Interestingly, vagal stimulation reduced gut barrier permeability after TBI, mediated by the suppression of TNF-α release (106). Recently, it has been demonstrated that FMT can restore gut microbiota dysbiosis following TBI and ameliorate neurological deficits, mediated by the TMA-TMAO-MsrA signaling pathway (96).

### Spinal Cord Injury (SCI)

Traumatic spinal cord injury (SCI) is another acute CNS injury that affects millions worldwide every year (107). The studies involving SCI and the bidirectional effect on the gut microbiota have been carried out in recent years, which are summarized in **Table 3**. This section reviews the study published on the changes in the gut microbiota that occur following SCI.

**Table 3:** A summary of preclinical and human studies on the gut microbiota and spinal cord injury.

	Subjects	Methods	Key findings
Gungor B, et al. (2016) (108)	30 patients vs. 10 controls	16S rRNA (V4) sequencing	Marvinbryantia was significantly lower in the upper motor neuron (UMN) bowel dysfunction group than in the lower motor neuron (LMN) group after spinal cord injury(SCI). Compared with healthy groups, Roseburia, Pseudobutyrivibrio, and Megamonas were significantly lower in the LMN bowel dysfunction group; the abundances of Pseudobutyrivibrio, Dialister, and Megamonas genera were significantly lower in the UMN bowel dysfunction group.
Zhang C, et al. (2018) (109)	43 patients vs. 23 controls	16S rRNA (V3-V4) sequencing	SCI contributed to the increased abundance of Veillonellaceae and Prevotellaceae and the decreased abundance of Bacteroidaceae and Bacteroides. The abundance of Bacteroidaceae and Bacteroides in the quadriplegia group and Acidaminococcaceae, Blautia, Porphyromonadaceae, and Lachnoclostridium in the paraplegia group were significantly higher compared to the healthy male group. Following SCI, the gut microbiota composition was significantly associated with serum biomarkers (glucose, high-density lipoprotein, creatinine, and C-reactive protein), neurogenic bowel dysfunction defecation time, and course.
Lin R, et al. (2020) (110)	23 patients vs. 23 controls	16S rRNA (V3-V4) sequencing	There were no significant differences in the α-diversity indices of the fecal microbiota between the SCI and control groups. The abundances of Parabacteroides, Alistipes, Phascolarctobacterium, Christensenella, Barnesiella, Holdemania, Eggerthella, Intestinimonas, Gordonibacter, Bilophila, Flavonifractor, and Coprobacillus were higher in the patients with SCI than those in the healthy control.
Li J, et al. (2020) (111)	32 patients (7 acute SCI and 25 long-standing SCI) vs. 25controls	16S rRNA (V4) sequencing	Compared with the controls, SCI patients had higher abundances of the Erysipelotrichaceae, Acidaminococcaceae, Rikenellaceae, Lachnospiraceae, Rikenellaceae, the Ruminococcaceae families. The long-standing SCI patients had higher abundances of Lachnospiraceae and Eggerthellaceae, and lower abundances of Campylobacteraceae than the controls. The acute SCI has a higher abundance of the Desulfovibrionaceae family than the controls.
Bazzocchi G, et al. (2021) (112)	100 patients	16S rRNA (V3-V4) sequencing	The SCI-induced gut microbiota composition showed distinct dysbiotic signatures with an enriched in potentially pathogenic, pro-inflammatory, and mucus-degrading bacteria and a decreased abundance of short-chain fatty acids (SCFAs) producers. The gut microbiota dysbiosis is very likely secondary to injury and closely related to the lesion’s degree of completeness and severity after SCI.
Yu B, et al. (2021) (113)	45 patients (21 complete thoracic SCI and 24 incomplete thoracic SCI) vs. 24 controls	16S rRNA sequencing	Compared with healthy individuals, Actinobacteria and Synergistetes were significantly enriched in patients with complete thoracic SCI. Similarly, Bacteroidetes, Cyanobacteria, and Proteobacteria were significantly lower in patients with incomplete thoracic SCI than healthy controls. Coriobacteriaceae, Synergistetes, Eubacterium, and Cloacibacillus, were significantly increased in patients with complete thoracic SCI, while Lactobacillaceae, Lachnospiraceae, Eubacterium, Clostridium, and Sutterella, were significantly increased in patients with incomplete thoracic SCI.
Kigerl KA, et al. (2016) (25)	T9 contusion mice model	16S rRNA (V4-V5) sequencing	SCI increased intestinal permeability and bacterial translocation from the gut, associated with immune cell activation in gut-associated lymphoid tissues (GALTs) and the altered gut microbiota composition. In naive mice, gut dysbiosis induced by oral delivery of broad-spectrum antibiotics before SCI exacerbated neurological impairment and spinal cord pathology after SCI. SCI mice treated by commercial probiotics (VSL#3) enriched lactic acid-producing bacteria, triggering a protective immune response in GALTs and conferring neuroprotection with improved neurological recovery.
O’Connor G, et al. (2018) (114)	T9 contusion rat model	16S rRNA (V4) sequencing	Lactobacillus intestinalis, Clostridium disporicum, and Bifidobacterium choerinum were enriched, while Clostridium saccharogumia was depleted following SCI. Levels of interleukin-1β(IL-1β), IL-12, and macrophage inflammatory protein-2 significantly correlated with changes in β diversity 8-weeks post-SCI.
Myers SA, et al. (2019) (115)	T9 contusion mice model	16S rRNA (V4) sequencing	SCI led to an increased abundance of Proteobacteria. The absence of Pde4b improved white matter sparing and recovery of hindlimb locomotion following SCI. Moreover, SCI-induced gut dysbiosis, bacterial overgrowth, and endotoxemia were also reduced in Pde4b^-/-^ mice.
Jing Y, et al. (2019) (116)	T10 contusion mice model	16S rRNA (V3-V4) sequencing	Daily intraperitoneal injection with melatonin improved enhanced barrier integrity and gastrointestinal motility, reduced proinflammatory cytokines, and promoted locomotor recovery. Melatonin-treated SCI animals decreased the abundance of Clostridiales and increased the quantity of Lactobacillales and Lactobacillus. Before surgery, gut dysbiosis induced by broad-spectrum antibiotics exacerbated neurological impairment following SCI, and melatonin treatment improved locomotor recovery and intestinal integrity in antibiotic-treated SCI mice.
Schmidt EKA, et al. (2020) (117)	C5 contusion rat model	16S rRNA (V4) sequencing	SCI-induced dysbiosis increased symptoms of anxiety-like behavior. Fecal microbiota transplantation (FMT) prevented SCI-induced dysbiosis and the development of anxiety-like behavior.
Jing Y, et al. (2021) (118)	T10 contusion mice model	16S rRNA (V4) sequencing & HPLC-MS	FMT-treated SCI mice facilitated functional recovery, promoted neuronal axonal regeneration, and enhanced intestinal barrier integrity and gastrointestinal motility, which short-chain fatty acids (SCFAs) and Nuclear Factor-κB (NF-κB) signaling may mediate. Butyricimonas were reduced in SCI mice, and FMT significantly reshaped gut microbiota.
Schmidt EKA, et al. (2021) (119)	C5 contusion rat model	16S rRNA (V4) sequencing	Minocycline had a profound acute effect on the gut microbiota diversity and composition after SCI. Gut dysbiosis following SCI has been linked to the development of anxiety-like behavior, which was also alleviated by minocycline. Although minocycline attenuated SCI-induced microglial activation, it did not change the lesion size or promote neurological recovery.
Doelman A, et al. (2021) (120)	T2 or T10 contusion pig model	16S rRNA (V3-V4) sequencing	In the acute phase (<14d post-SCI), Proteobacteria, Tenericutes, Epsilonbacteraeota, and Cyanobacteria decreased compared to the controls while Bacteroidetes, Firmicutes, and Spirochaetes were enriched. In the sub-acute phase (>14 days post-SCI), the abundance of Spirochaetes, Cyanobacteria, and Proteobacteria remained statistically significantly different from the controls.
Rong Z, et al. (2021) (121)	T10 contusion mice model	–	The levels of pro-inflammatory cytokines tumor necrosis factor-α, IL-1β, and IL-6 in SCI mice were increased, while the levels of anti-inflammatory factors IL-4, transforming growth factor-β, and IL-10 were decreased. Gut microbiota dysbiosis aggravated SCI by activating the toll-like receptor 4(TLR4)/myeloid differentiation factor 88 (MyD88) signaling pathway.
Du J, et al. (2021) (122)	T4 or T10 contusion mice model	genome- and gene-resolved metagenomic analysis	The abundance of Lactobacillus johnsonii and CAG-1031 spp. decreased, while Weissella cibaria, Lactococcus lactis_A, Bacteroides thetaiotaomicron were enriched after SCI. Microbial-mediated biosynthesis of tryptophan, vitamin B6, and folate was reduced after SCI. 1028 mostly novel viral populations were recovered, which expanded known murine gut viral species sequence space. Phages of beneficial commensal hosts, including CAG-1031, Lactobacillus, and Turicibacter, decreased, while phages of pathogenic hosts, including Weissella, Lactococcus, and class Clostridia, increased after SCI.

16S rRNA, 16S ribosomal RNA.

HPLC-MS, high-performance liquid chromatography-mass spectrometry.

In a Chinese cohort study, Zhang et al. observed an increase in Proteobacteria and Verrucomicrobia and reduced Bacteroidaceae and Bacteroides in patients with chronic traumatic complete SCI (109). Lin et al. also analyzed 46 Chinese subjects (23 SCI patients vs. 23 healthy controls) and reported that the abundances of Parabacteroides, Alistipes, Phascolarctobacterium, Christensenella, Barnesiella, Holdemania, Eggerthella, Intestinimonas, Gordonibacter, Bilophila, Flavonifractor, and Coprobacillus were higher in the patients with SCI than those in the health individuals (110). Another clinical study with 54 Turkish participants (41 SCI patients vs. 13 healthy controls) identified that butyrate-producing microbes of the Firmicutes phylum are significantly reduced in SCI patients than healthy controls (108). Recently, Bazzocchi et al. investigated a large Italian SCI population acute phase after injury and age- and gender-matched healthy Italians (112). Their study revealed that the abundance of SCI patients’ gut microbiota increased in potentially pathogenic, pro-inflammatory, and mucus-degrading bacteria and decreased in SCFAs producers. Moreover, gut microbiome dysbiosis is closely associated with the severity of the lesion after SCI. A case-control study carried by Yu et al. (45 SCI patients vs. 24 healthy individuals) showed that the abundance of Actinobacteria and Synergistetes in patients with complete thoracic SCI (CTSCI) was significantly higher than that in healthy individuals. At the same time, the Bacteroidetes, Cyanobacteria, and Proteobacteria were significantly decreased in patients with incomplete thoracic SCI (ITSCI) as compared to the healthy (113). Furthermore, they compared the gut microbiota composition between patients with CTSCI and ITSCI and found a significantly increased abundance of Coriobacteriaceae, Synergistetes, Eubacterium, and Cloacibacillus was observed in patients with CTSCI, while patients with ITSCI were abundant with Lactobacillaceae, Lachnospiraceae, Eubacterium, Clostridium, and Sutterella.

Similarly, a thoracic level 9 (T9) contusion SCI-induced gut microbiota dysbiosis in the experimental SCI mice was also characterized by an expansion of Bacteroidetes and a reduction of Firmicutes (115). However, a preclinical work in a T9 contusion SCI mouse model by Kigerl et al. reported that SCI mice presented a decrease in Bacteroidales and an increase in Clostridiales (25). In an SCI rat model, gut microbiota composition was significantly changed with an increased abundance of Lactobacillus intestinalis, Clostridium disporicum, and Bifidobacterium choerinum and a reduced level of Clostridium saccharogumia (114). The difference in the results may be caused by experimental deviation. Additionally, the above analyses of SCI-induced gut microbiota dysbiosis were assessed by 16S rRNA amplicon sequencing, which cannot profile microbiota function or identify viruses (123). Du et al. studied gut microbiota dysbiosis after experimental SCI at T4 or T10 using genome- and gene-resolved metagenomic analysis (122). The results suggested that the abundance of beneficial commensals (Lactobacillus johnsonii and CAG-1031 spp.) significantly decreased, while potentially pathogenic bacteria (Weissella cibaria, Lactococcus lactis_A, Bacteroides thetaiotaomicron) increased after SCI. Functionally, tryptophan, vitamin B6, and folate biosynthesis, encoded by microbial genes, were reduced in the feces after SCI. Interestingly, the study performed by Du et al. reported that phages of beneficial commensal hosts (CAG-1031, Lactobacillus, and Turicibacter) decreased. In contrast, phages of pathogenic hosts (Weissella, Lactococcus, and class Clostridia) increased after SCI (122). In a Yucatan minipig model with a contusion-compression SCI at T2 or T10, Doelman et al. presented a dynamic view of the microbiome changes following SCI and identified acute stage, 0-14 post-SCI, as a special time-frame that many of the bacterial fluctuations occur before returning to “baseline” levels (120).

SCI promotes intestinal leakiness and bacterial translocation associated with activation of immune cells in GALTs, by increasing the population of B cells, CD8^+^ T cells, DCs, and macrophages and decreasing CD4^+^ T cell counts (25). γδT cell-deficient mice improved functional recovery after SCI (124). Moreover, changes in gut microbiota composition following SCI could predict locomotor impairment (125). Additionally, gut microbiota dysbiosis can aggravate SCI by activating the TLR4/Myeloid differentiation factor 88 signaling pathway (121).

SCI mice fed with commercial probiotics (VSL#3) reduced neuropathology, improved locomotor recovery, and promoted an anti-inflammatory response by increasing the number of Treg cells in GALTs (25). Additionally, SCI mice daily treated with melatonin improved gut barrier integrity and functional recovery by reducing the abundance of Clostridiales and enhancing the quantity of Lactobacillales and Lactobacillus, which were related to a more favorable cytokine profile (116). Lactic acid supplementation was also proved to improve functional recovery following SCI (25). FMT prevented both SCI-induced dysbiosis, locomotor function, and the development of anxiety-like behavior (117). FMT could increase the amount of fecal SCFAs and downregulate IL-1β/NF-κB signaling in the spinal cord and NF-κB signaling in the gut following SCI (118). A recent study also reported that minocycline treatment attenuated SCI-induced anxiety-like behavior and systemic inflammatory response *via* altering the Firmicutes/Bacteroidetes ratio (119). Engineered liposomes targeting the MGBA may also be a potential treatment (126).

## Conclusion and Perspective

Gut microbiota is closely involved in the development and progression of acute CNS disease through multiple mechanisms, including immunological, endocrine, metabolic, and neural pathways. FMT and probiotics significantly improve brain injury by restoring the acute CNS injury-induced gut microbiota dysbiosis. Gut microbiota may be a potential target to assist in the treatment of acute CNS injury. However, several aspects are still needed to ponder despite a growing number of studies concerning the gut microbiota. Firstly, human gut microbiota composition is different from rodents. Although Firmicutes and Bacteroidetes are the most abundant microbiota both in mice and humans, more than 80% of the bacteria found in the mice intestine are not colonized in the human intestines based on genus level (127). Secondly, immunological features are also different between rodents and humans. A previous study suggests that the intestinal properties of humans are similar to those of mice. However, differences in intestinal immunity between mice and humans have already been found that γδT cells are found significantly less frequently in the intraepithelial compartment of humans than in mice (128). Thirdly, the effects of enteroviruses, fungi, and bacteriophages cannot be ignored. Bacteriophages have high host specificity that shapes the gut microbiota composition and regulates the host immune response by altering bacterial pathogen-associated molecular patterns and maintaining the host mucosal barrier (129). Although the effects of enteroviruses on health and disease are still unclear, phage-virus-fungi-bacterial-host interaction in the gut should also be considered. Moreover, their role in human acute brain injury or animal models has not been studied so far. Fourthly, developmental disturbances in GF mice should be considered. GF mice have hypoplastic immune structures and differ from SPF mice in the intestinal immune cell populations, such as IgA-producing plasma cells and lamina propria CD4^+^ T cells (130). Additionally, GF mice contain fewer serum immunoglobulins, particularly IgG (131). In the absence of gut microbiota, CNS is also altered with a “leaky” BBB and an abnormal microglia morphology and function (132, 133). Finally, criteria for identifying qualified, healthy donors in the FMT treatment have not yet been fully established. The safety and efficiency of FMT need to be extensively investigated.

## Author Contributions

BY wrote the manuscript. X-jL and QW revised the manuscript. All authors contributed to the article and approved the submitted version.

## Funding

This work was supported by the Scientific Research Project of Jiangsu Provincial Health commission under Grant No. H2018066.

## Conflict of Interest

The authors declare that the research was conducted in the absence of any commercial or financial relationships that could be construed as a potential conflict of interest.

## Publisher’s Note

All claims expressed in this article are solely those of the authors and do not necessarily represent those of their affiliated organizations, or those of the publisher, the editors and the reviewers. Any product that may be evaluated in this article, or claim that may be made by its manufacturer, is not guaranteed or endorsed by the publisher.
